# Polymorphic variants conferring genetic risk to cervical lesions support *GSTs* as important associated loci

**DOI:** 10.1097/MD.0000000000017487

**Published:** 2019-10-11

**Authors:** Sijuan Tian, Xiaofeng Yang, Li Zhang, Juan Zhao, Meili Pei, Yang Yu, Ting Yang

**Affiliations:** Department of Obstetrics and Gynecology, the First Affiliated Hospital of Xi’an Jiaotong University, Xi’an, Shaanxi, China.

**Keywords:** cervical lesions, *GSTM1*, *GSTP1*, *GSTT1*, polymorphisms

## Abstract

To analyze the association between glutathione S-transferases polymorphisms and the risk of cervical lesions.

Case-control studies focusing on the association between glutathione S-transferase polymorphisms and the risk of cervical lesions were collected from the PubMed, Web of Science, Cochrane Library, Embase, Medline, CNKI, VIP and Wanfang databases from inception to August 2018. Pooled odds ratios and 95% confidence intervals were employed to evaluate the strength of the association. Subgroup analysis and sensitivity analysis were used to test the potential discrepancy and robustness, respectively.

A total of 30 studies comprising 3961 patients and 4726 healthy controls satisfied the inclusion criteria. Of these, 6 studies contained information about *GSTP1*, 27 studies contained information about *GSTM1*, and 22 studies contained information about *GSTT1*. Our results supported that there was no statistical association between *GSTP1* polymorphism and the risk of cervical lesions (odds ratio [OR] = 1.08, *P* = .40). The *GSTM1* null variant showed increased susceptibility to cervical lesions (OR = 1.45, *P* < .001). Subgroup analysis revealed that the *GSTM1* null variant caused cervical lesions among HPV infection cases (OR = 1.69, *P* = .02) and among the Chinese and Indian populations (OR = 2.24 and OR = 1.87, respectively, *P* < .001). The *GSTT1* null variant increased the risk of cervical lesions in smokers (OR = 1.52, *P* = .03). The *GSTT1* null genotype was also related to high-grade intraepithelial neoplasia (HSIL) and cervical cancer risk (OR = 1.30 and OR = 1.78, respectively, *P* < .05).

The *GSTM1* null variant caused cervical lesions, especially among HPV infection cases and among the Chinese and Indian populations. The *GSTT1* null variant increased the risk of cervical lesions in smokers and was also related to HISL and cervical cancer risk.

## 1 Introduction

Cervical cancer ranks fourth for both incidence and mortality rates in women, with an estimated 570,000 cases and 311,000 deaths in 2018 worldwide. In lower human development index (HDI) regions, it is the second most frequently diagnosed cancer and the second leading cause of cancer death.^[1]^ In China, the results indicated that an estimated 98,900 new cases and 30,500 cancer deaths occurred in 2015.^[2]^ Human papillomavirus (HPV) is considered a major factor in cervical cancer. Other co-factors are also important in cervix carcinogenesis, including immune suppression, cigarette smoking, parity, and oral contraceptive use.

Glutathione S-transferases (GSTs) are a family of phase II enzymes that are responsible for the metabolism of various xenobiotics and carcinogens by catalyzing the conjugation of glutathione to electrophilic compounds.^[3]^ Studies have shown that genetic variations in *GSTs* affect human phase II detoxification enzymes, thereby altering their ability to detoxify various exogenous and endogenous active species.^[4]^

Previous studies revealed that the *GST* genetic variants were related to the risk of several cancers, such as breast, lung, prostate, bladder, and nasopharyngeal cancer risk.^[5]^ However, the results were controversial regarding whether *GST* polymorphisms would lead to the development of cervical lesions, so we conducted this meta-analysis about the relationship between *GST* genetic variants and cervical lesions risk.

## 2 Material and methods

### 2.1 Literature search strategy

We searched the Cochrane Library, Embase, Medline, PubMed, Web of Science, CNKI, Wanfang, and VIP databases by the following search terms: Glutathione Transferase[Mesh] or GST∗, glutathione S-transferase pi[Mesh] or GSTP1, glutathione S-transferase M1[Mesh] or GSTM1, glutathione S-transferase T1[Mesh] or GSTT1, polymorphism∗/variant∗/mutation∗/SNP, Uterine Cervical Neoplasm [Mesh]/cervix cancer/cervical cancer/cervical neoplasm∗/cervical carcinoma∗, and the combinations of these. In addition, we searched the reference lists of all identified articles manually to acquire more data.

### 2.2 Inclusion and exclusion criteria

Studies included needed to meet the following criteria: regarding on the association between *GST* gene polymorphisms (*GSTP1/GSTM1/GSTT1*) and the risk to cervical lesions; human study subjects; case-control studies; available and sufficient genotype distribution data to calculate odds ratios (ORs) and corresponding 95% confidence intervals (CIs); and diagnoses based on cervical biopsy pathology or cytology. Besides, if there were duplicate studies, the most complete one was reserved. Otherwise, the article was excluded if it did not satisfy the criteria above.

### 2.3 Data extraction and synthesis

Two investigators extracted relevant data from all the eligible studies independently. A third reviewer was invited to participate in the work when some disagreement occurred; consensus was ultimately reached by discussion. According to the 4th WHO Women's Genital Tumor Classification Guidelines, we defined cervical lesions as cervical cancer, high-grade intraepithelial neoplasia (HSIL), and low-grade intraepithelial neoplasia (LSIL). LSIL was equivalent to cervical intraepithelial neoplasia (CIN) grade 1, and HSIL included most amount of CIN2 and all CIN3 cases.^[6]^ We gathered characteristics from all satisfied records: the first author, publication year, ethnicity, total numbers of cases and controls, source of controls, genotyping method.

### 2.4 Statistical analysis

Using the ORs and 95% CIs to assess the degree of association between *GSTs* polymorphic variants and cervix lesions. A Z-test revealed statistical significance when *P* < .05. *I*^2^ and *Q* statistic were applied to detect heterogeneity among different studies. There was no heterogeneity if *I*^2^ < 50% and *P* > .1 and a fixed effect model was used, otherwise we thought heterogeneity existed in the incorporated populations and a random effect model was used instead. Subsequently, we conducted a subgroup analysis according to HPV infection status, cigarette smoking, degree of cervix lesions, and ethnicity. Hardy-Weinberg equilibrium (HWE) was evaluated by chi-square test with *P* < .05 indicating a deviation from HWE. Sensitivity analysis was employed to estimate stability of the meta-analysis results by deleting all the studies one by one. Additionally, a Begg funnel plot and an Egger test were used to evaluate publication bias. The statistical analyses were performed using RevMan 5.3 (Cochrane Collaboration) and STATA 12.0 (StataCorp., College Station, TX, USA) software.

## 3 Results

### 3.1 Characteristics of included studies

**Figure 1:**
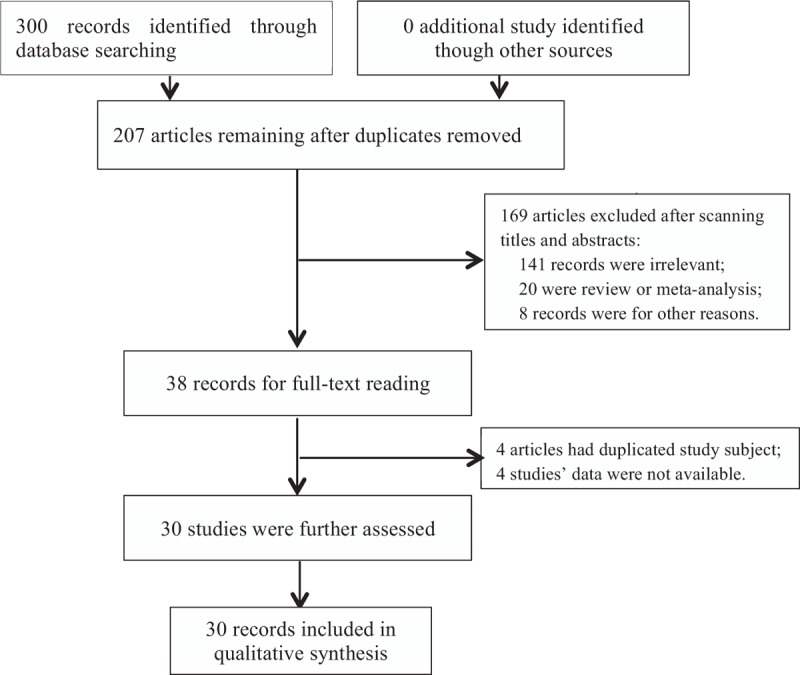
Flow diagram of searching procedure.

By searching the electronic databases systematically, we initially retrieved 300 articles. After excluding duplicate studies, 207 articles remained. Further reviewing of the titles and abstracts of the identified studies allowed the removal of 169 articles. Of those removed, 141 were clearly irrelevant to *GST* polymorphisms, 20 were review papers or meta-analyses, 8 records were deleted for other reasons. We downloaded the remaining 38 articles as full-text reports and reviewed them carefully. Four records were excluded for containing duplicate samples, and the data were not available in other 4 studies. Finally, 30 case-control studies containing 3961 cases and 4726 controls were included, among which 6 studies were about *GSTP1*, 27 articles were on *GSTM1*, and 22 studies focused on *GSTT1* (Fig. 1). The characteristics of included studies were presented at Table 1.

**Table 1:**
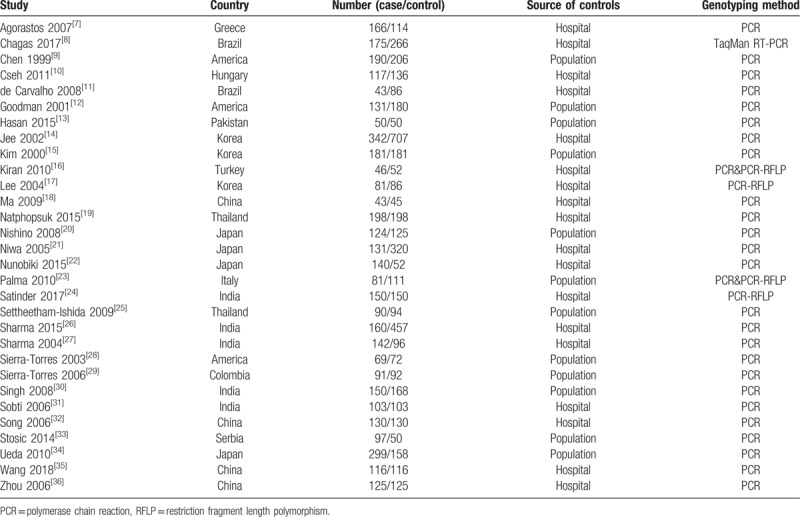
Characteristics of the included studies.

### 3.2 Meta-analysis results

**Figure 2:**
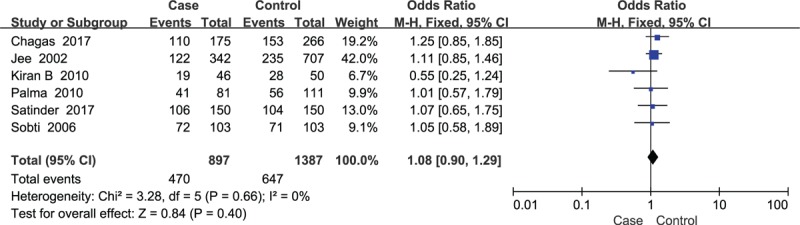
Forest plots of the association between *GSTP1* polymorphism and susceptibility of cervical lesions in dominant genetic model.

There were 6 studies on the *GSTP1* variant that included 897 cases and 1387 healthy controls. The meta-analysis results did not show a statistical association between *GSTP1* polymorphism and the risk of cervical lesions in the dominant genetic model (OR = 1.08, *P* = .40) (Fig. 2).

**Figure 3:**
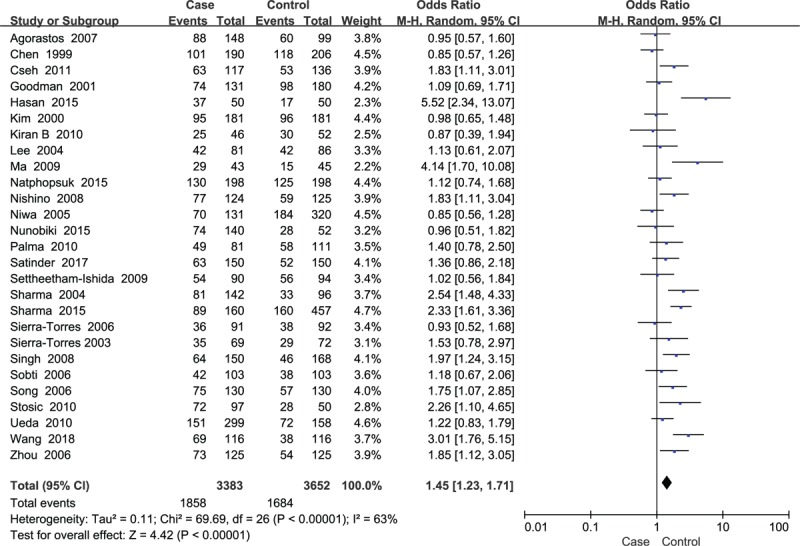
Forest plots of the association between *GSTM1* polymorphism and susceptibility of cervical lesions.

**Figure 4:**
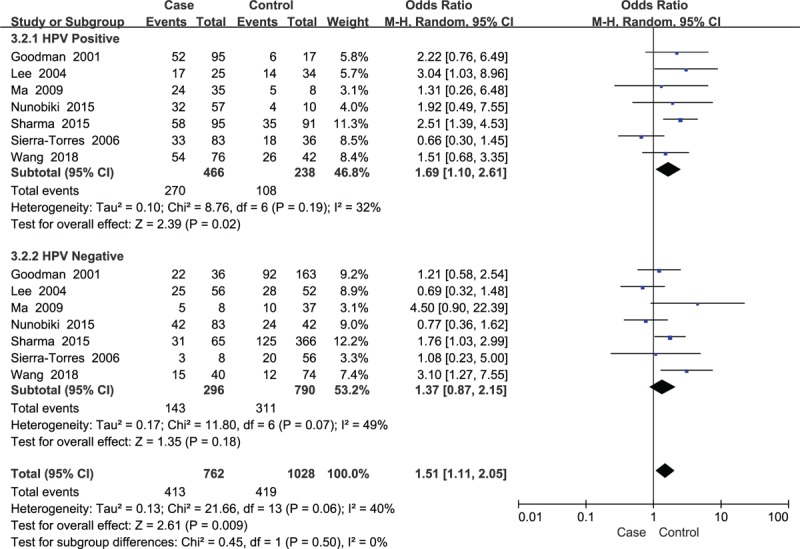
Subgroup analysis of the association between *GSTM1* polymorphism and cervical lesions stratified by HPV infection status. HPV = human papillomavirus.

A total of 27 case-control studies were included in the meta-analysis of *GSTM1* involving 3383 cases and 3652 controls. The results showed that the *GSTM1* null allele was related to an increased risk of cervical lesions (OR = 1.45, *P* < .001) (Fig. 3). Great heterogeneity existed in the *GSTM1* studies (*P* < .001, *I*^2^ = 63%), thus, a random-effect model was employed. In addition, we conducted subgroup analysis based on HPV infection status, smoking status, degree of cervical lesions, ethnicity. The results presented in Table 2. The *GSTM1* null variant was related to an increased risk of cervical lesions among HPV positive cases (OR = 1.69, *P* = .02) (Fig. 4), nonsmokers (OR = 1.73, *P* < .001), and Chinese and Indian populations (OR = 2.24 and OR = 1.87, respectively, *P* < .001), but was not related to the degree of cervical lesions (Table 2).

**Table 2:**
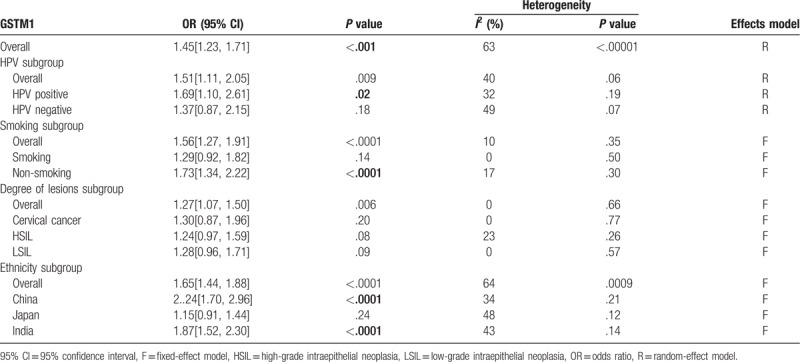
Meta-analysis results of *GSTM1* polymorphism.

**Figure 5:**
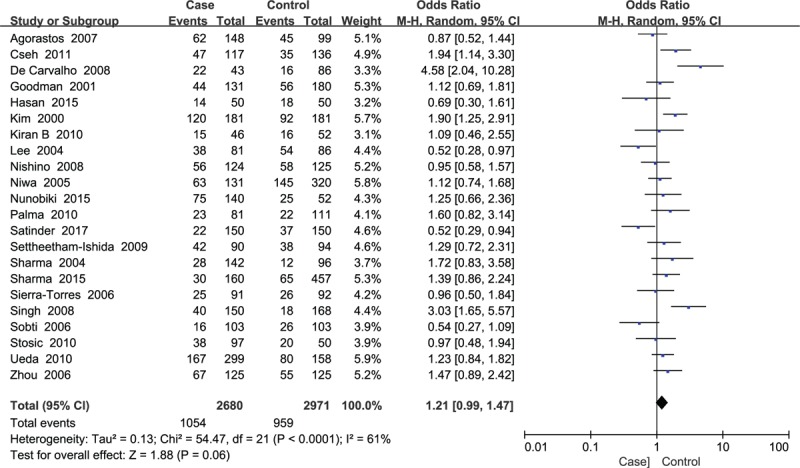
Forest plots of the association between *GSTT1* polymorphism and susceptibility of cervical lesions.

**Figure 6:**
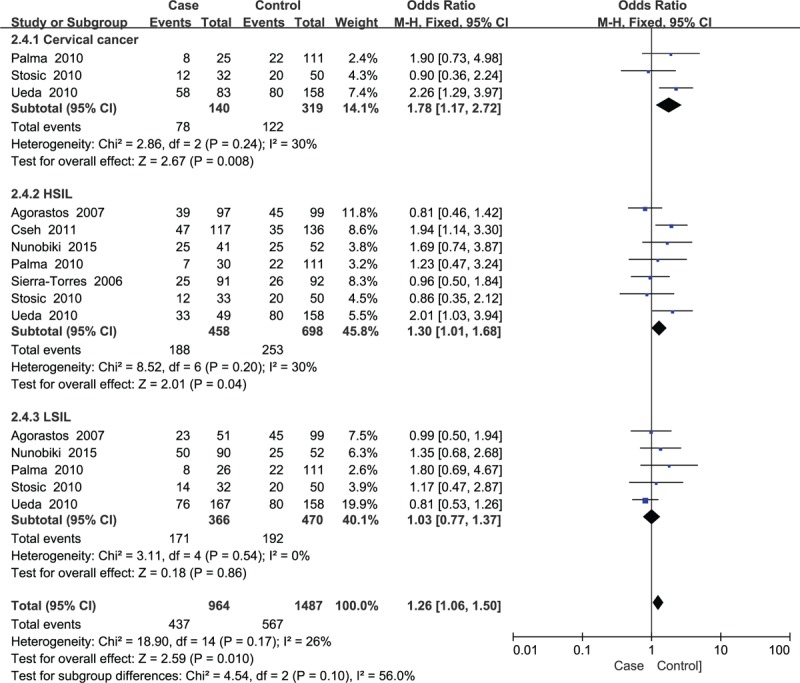
Subgroup analysis of the association between *GSTT1* polymorphism and cervical lesions stratified by degree of lesions.

For the *GSTT1* genotype, there were 2680 cases and 2971 controls incorporated in the study. The pooled OR suggested that the *GSTT1* null genotype might not be related to cervical lesions (*P* = .06) (Fig. 5). Considering the heterogeneity, we performed a subgroup analysis stratified by HPV infection status, cigarette smoking, degree of cervical lesions, and ethnicity. The results revealed that the *GSTT1* null variant increased cervical lesions in smokers (OR = 1.52, *P* = .03). In addition, the *GSTT1* null variant was related to HISL and cervical cancer (OR = 1.30 and OR = 1.78, respectively, *P* < .05) but was not related to LSIL (Fig. 6). HPV infection status and ethnicity did not modify the association between *GSTT1* polymorphism and cervical lesions (Table 3).

**Table 3:**
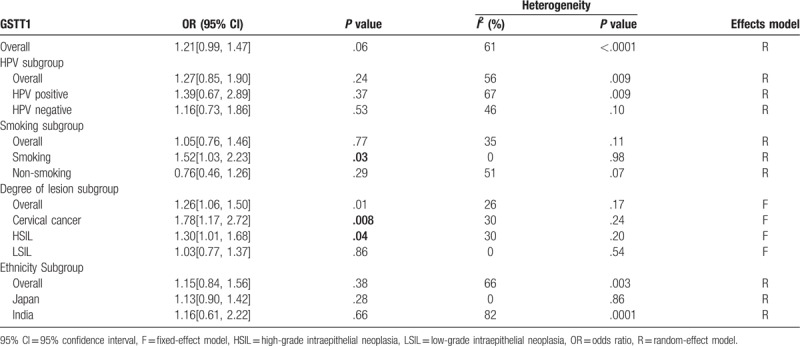
Meta-analysis results of *GSTT1* polymorphism.

### 3.3 Detection for heterogeneity and sensitivity analysis

As presented in Tables 2 and 3, there was great heterogeneity among studies relating to *GST* genetic variants (*I*^2^ > 50%, *P* < .1). In consideration of this, we used a random effect model for the meta-analysis. Additionally, subgroup analysis stratified by HPV infection status, cigarette smoking, degree of cervical lesions, and ethnicity was performed to eliminate heterogeneity. Heterogeneity was clearly decreased in the ethnicity subgroup. This indicated that ethnicity might be a confounding factor and heterogeneity source, while the pooled ORs were substantially robust.

**Figure 7:**
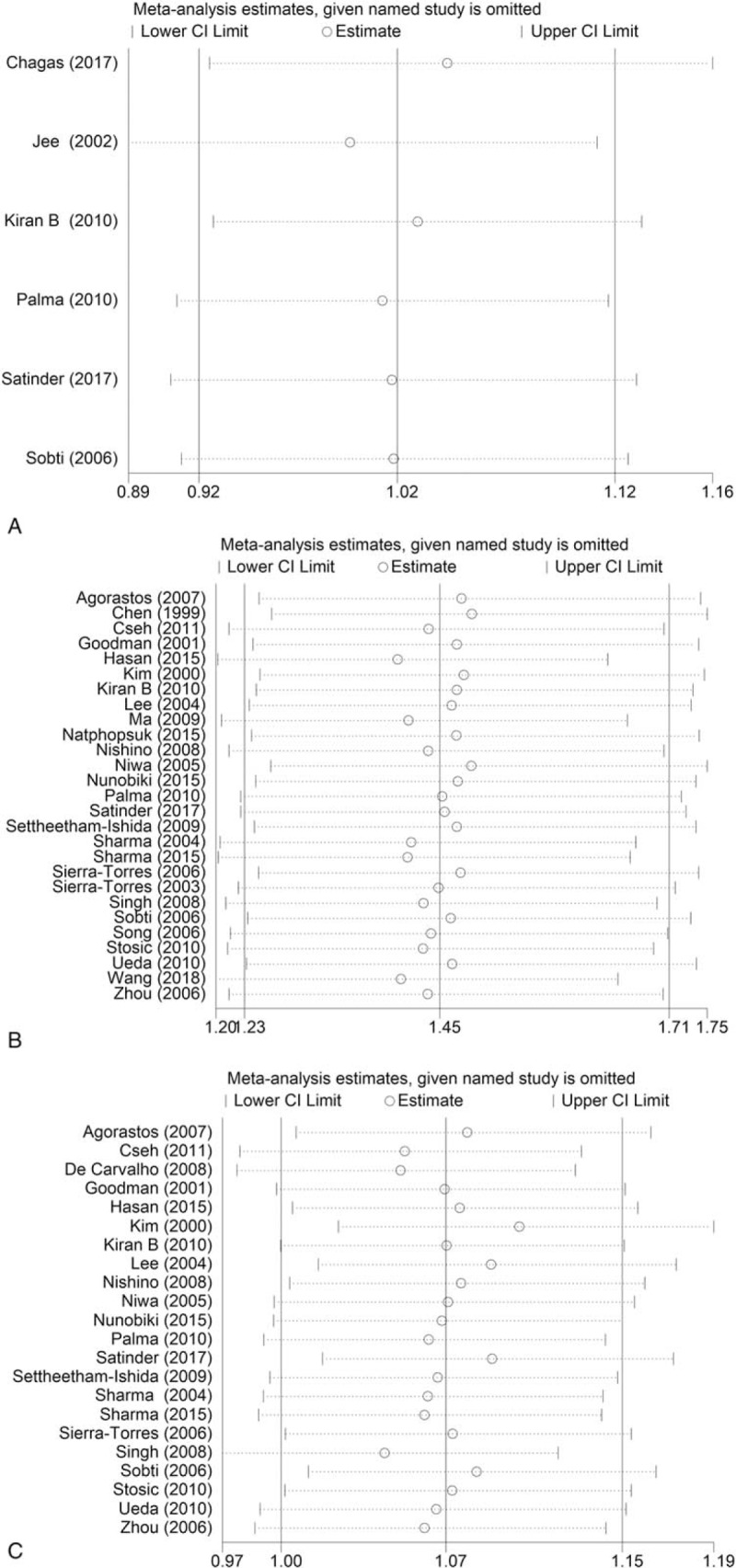
Sensitivity analysis of the association between *GST* SNPs and risk of cervical lesions. (A) *GSTP1*; (B) *GSTM1*; (C) *GSTT1*.

Sensitivity analysis was utilized to evaluate the stability of the meta-analysis by deleting all the studies one by one. The pooled ORs did not change significantly in any of the *GST* variants, indicating that the meta-analysis was robust and stable (Fig. 7).

### 3.4 Publication bias

**Figure 8:**
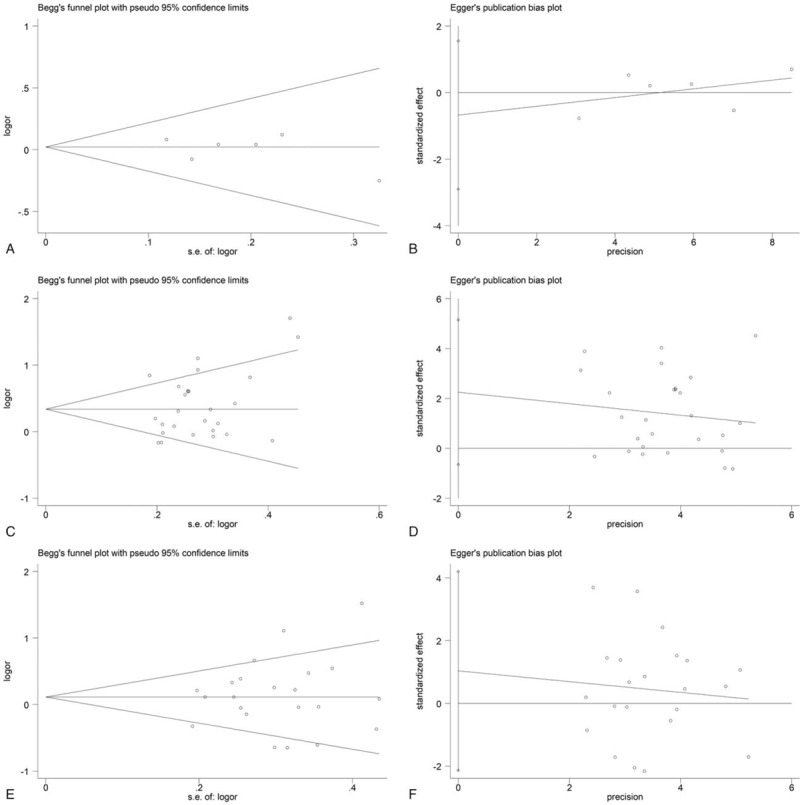
Publication bias of *GST* polymorphisms. (A, B). *GSTP1*, Begg test, *P* = .452, Egger test, *P* = .448; (C, D). *GSTM1*, Begg test, *P* = .144, Egger test, *P* = .122; (E, F). *GSTT1*, Begg test, *P* = .778, Egger test, *P* = .502.

To detect publication bias, Begg funnel plot and Egger test were performed. The results indicated that no significant evidence of publication bias for *GSTP1*, *GSTM1*, and *GSTT1* variant was observed in our study (*P* > .05) (Fig. 8).

## 4 Discussion

Cervical cancer is an outcome of virus-induced carcinogenesis. HPV is the primary etiology of cervical carcinogenesis but all HPV infections do not result in cervical cancer. Tobacco use, immune system function, use of oral contraceptive, number of sexual partners all modify the outcome of cervix lesions.

GSTs play an important role in protecting cells from oxidative damage and in modulating the induction of other enzymes and proteins in response to DNA damage, therefore, they are important for maintaining genomic integrity.^[37]^ GSTs catalyzed the conjugation of glutathione to electrophilic substrates, which resulted in the enhanced renal clearance and reduced carcinogenic load from the cell.^[38]^

The *GSTP1* G/A single nucleotide polymorphism caused valine (Val) took the place of isoleucine (Ile) at codon 105, resulting in decreased enzymatic activity and low ability to metabolize certain xenobiotics and carcinogens.^[39]^ Biochemical studies indicated that the *GSTP1* AA genotype was 2 to 3 times less stable^[40]^ and might be associated with the risk of gynecological cancer. However, our results supported that *GSTP1* AA genetic variant was not associated with the risk of cervix lesions, which was consistent with Zhao finding.^[38]^ This might be attributed to an insufficient sample size.

With regard to the *GSTM1* and *GSTT1* genotypes, some studies indicated that the *GSTM1* null or *GSTT1* null variants contributed to cervical cancer susceptibility, while some studies showed that the 2 variants were not associated with cervical carcinogenesis. Our results supported that the *GSTT1* null variant increased the risk of cervical lesions in smokers. The *GSTT1* null genotype was also related to HISL and cervical cancer risk. The *GSTM1* null variant increased susceptibility to cervical carcinogenesis. Subgroup analysis revealed that the *GSTM1* null variant caused cervical lesions among HPV infection cases and among the Chinese and Indian populations. This implied that there were differences in ethnicity and environment. In addition, it elevated the risk of cervical lesions among women who were not smoking, which implied that the *GSTM1* null genotype might be a risk factor independent of cigarette smoking.

A previous study demonstrated that the *GST* null genotype resulted in complete loss of the ability of the enzyme to bind genotoxic substrates. This leads to decreased detoxification ability, a reduction in the metabolic rate of intracellular toxic substances, and increased malignant transformation of cells, which thereby promoted tumorigenesis.^[40]^ Several studies on the relationship between *GST* polymorphisms and cervical cancer risk were conducted. Compared with those studies, our meta-analysis included additional qualified studies to evaluate the association and therefore obtained more persuasive conclusions. Additionally, the study included the association of *GSTP1*, *GSTM1*, and *GSTT1* genetic variants on cervical lesion risk, while previous studies were based on only one or two of the three variants. Moreover, to eliminate the effects of co-factors, we performed subgroup analysis stratified by HPV infection status, cigarette smoking, degree of cervical lesion and ethnicity. Thus, our findings provide stronger evidence for the association between *GST* genetic variants and cervical lesions.

There are some limitations to our study. First, the small sample size was insufficient to support our results regarding the *GSTP1* genetic variant. Second, the incidence of cervical cancer is highest in sub-Saharan Africa, Latin America, the Caribbean, and Melanesia, where people of African origin account for the majority of the population.^[1]^ However, there were no statistics and studies of interest focused on women of African descent. This caused bias in the relationship, which is concerning. Additionally, although we considered the effect of age on our conclusions and attempted to perform a subgroup analysis, inconsistent age grouping of the included studies prevented us from conducting a subgroup analysis stratified by age. Last but not least, *GSTP1, GSTM1*, and *GSTT1* all belonged to the glutathione S-transferase family, playing an important role in protecting cells from oxidative damage and in metabolizing various carcinogens. As reported, the combination of the *GSTM1* null, *GSTT1* null, and *GSTP1* AA genotypes was associated with an increased risk of gynecological cancer, while the *GSTs* alone were not.^[23]^ Therefore, gene–gene interactions are likely more appropriate to assess disease risk than individual genes. In our meta-analysis, there was no association study between gene–gene interactions and the risk of cervical lesions. Future studies containing more comprehensive information are needed to obtain more reliable conclusions.

## 5 Conclusion

In general, the *GSTP1* AA genotype was not associated with the risk of cervical lesions. The *GSTM1* null variant caused cervix lesions, especially among HPV infection cases and among the Chinese and Indian populations. *GSTT1* null variant increased the risk of cervical lesions in smokers and was also related to HISL and cervical cancer risk. Additional large, well-designed case-control studies are needed to authenticate these results.

## Author contributions

**Conceptualization:** Sijuan Tian, Li Zhang, Ting Yang.

**Data curation:** Sijuan Tian, Xiaofeng Yang, Li Zhang, Juan Zhao, Meili Pei, Yang Yu, Ting Yang.

**Formal analysis:** Sijuan Tian, Xiaofeng Yang, Li Zhang, Juan Zhao, Yang Yu, Ting Yang.

**Funding acquisition:** Xiaofeng Yang, Juan Zhao, Ting Yang.

**Investigation:** Sijuan Tian, Xiaofeng Yang, Juan Zhao, Meili Pei, Yang Yu, Ting Yang.

**Methodology:** Sijuan Tian, Xiaofeng Yang, Juan Zhao, Meili Pei, Yang Yu, Ting Yang.

**Project administration:** Sijuan Tian, Xiaofeng Yang, Li Zhang, Ting Yang.

**Resources:** Ting Yang.

**Software:** Sijuan Tian, Ting Yang.

**Supervision:** Ting Yang.

**Validation:** Ting Yang.

**Visualization:** Sijuan Tian, Ting Yang.

**Writing – original draft:** Sijuan Tian, Ting Yang.

**Writing – review & editing:** Sijuan Tian, Ting Yang.
